# Supplementary material for: Quantitative analysis of chest computed tomography of COVID-19 pneumonia using a software widely used in Japan
Source: PLoS One. 2023 Oct 23;18(10):e0287953. doi: 10.1371/journal.pone.0287953 (PMC10593239; doi:10.1371/journal.pone.0287953)
Supplement: S1 Data — (PDF) [file pone.0287953.s001.pdf]

Clinical data

severity	age	sex	date from onset to CT	comorbidities	sympton	SpO2	WBC	lym%	CRP	LDH	AST	ALT	cre	eGFR
M	30	F	9	none	cough/fever/taste	99	3.5	26.6	1.22	211	26	25	0.56	102
M	48	F	12	none	cough/fever/taste	99	6.5	26	1.56	169	17	11	0.88	54
M	77	F	7	none	taste/diarrhea	97	2.7	25.9	0.74	253	29	16	0.51	86
M	19	F	5	none	cough/fever	97	8.5	4	0.5	156	14	7	0.6	1.6
M	73	F	1	none	fever	99	14.3	13.9	27.4	256	97	200	1.37	30
M	67	F	4	CRF,HT	cough/fever	99	3.9	12.6	0.87	201	17	10	9.54	4
M	74	F	1	none	fever/cough/diarrhea	96(3l)	4.3	8.6	3.12	222	20	22	0.65	67
M	84	F	6	none	fever	97(2l)	3.9	29.7	2.4	368	39	20	0.31	144
M	75	F	5	DM	fever/vomit	95	6.3	13.1	22.3	494	67	37	0.44	102
M	89	F	1	none	fever	95	21.7	5.3	24.4	240	37	15	0.9	44
M	58	M	3	none	none	98	8.6	14.2	2.25	299	25	17	1.06	57
M	47	M	3	none	cough/fever	97	5.9	19.5	4.35	301	30	20	1.09	58
M	49	M	2	none	fever/vomit	97	7.3	30.9	0.33	259	30	42	0.94	68
M	51	M	7	none	cough/fever	96	4.7	34.3	7.91	370	114	108	0.86	74
M	35	M	6	none	cough/fever/taste	100	4.3	21.6	0.33	238	26	15	0.84	85

M	22	M	10	none	fever/vomit	98	5.2	42	0.09	144	22	29	0.94	85
M	25	M	7	none	fever/taste	96	6	27.8	0.47	187	15	17	0.9.	86
M	55	M	6	none	fever/taste	96	4.3	26.1	1.94	390	36	27	0.99	62
M	60	M	1	none	cough/fever	96	8.4	17	9.62	287	29	30	0.68	91
M	36	M	9	none	cough/diarrhea	98	7.8	16.3	5.22	302	48	70	0.84	84
M	71	M	7	DM,HT	cough/fever	96	6.3	13.9	11.5	352	44	39	0.92	63
M	38	M	4	none	fever	97	3.6	46.5	0.02	210	36	40	0.95	72
M	41	M	11	none	cough	97	6.1	24.3	6.17	633	114	97	0.76	90
M	57	F	8	none	fever/taste/vomit	96	6.2	16.6	1.24	215	23	44	0.61	77
M	26	M	11	obs	fever	98	11.1	7	15.2	788	119	107	2.03	35
M	86	M	1	COPD	cough	96	3.9	13.9	2.62	190	36	9	0.84	65
M	69	M	1	none	fever	96	2.5	19.8	4.35	401	160	34	0.78	75
M	78	M	8	none	fever/taste	97	8.1	9.4	7.19	384	30	36	1.09	51
M	58	M	6	none	fever	97	5.3	13.6	4.8	272	55	52	1.21	49
M	88	M	1	COPD	fever	99(2l)	5.3	5.9	0.9	189	20	13	0.53	107
M	70	M	1	none	cough	95	3.7	27.8	1.36	262	22	20	1	57
M	47	M	9	none	fever	95	6.6	14.8	7	449	47	45	0.84	78
M	59	M	10	none	fever/diarrhea	95	6.7	19.2	12	485	91	88	0.66	95
M	76	M	1	cancer	fever	95	7.7	8.1	1.09	185	26	12	1	57
M	71	M	1	none	fever	95	4.2	28.4	3.33	259	29	29	1.08	52
M	89	M	1	none	fever	94	5.3	19.9	3.45	247	22	16	1.04	51
M	76	M	10	HT,CRF	cough/fever	95	3.3	14.3	3.28	314	132	85	2.31	22
M	66	M	1	DM,HT,CRF	cough/fever	94	4.9	9.2	29.6	324	15	15	14.9	3
S	80	M	12	none	cough/fever	94	5.3	18.2	2.43	233	25	15	0.76	74

S	44	M	4	obs	fever	95(3l)	7.5	20.6	5.3	615	51	33	1	65
S	58	M	11	obs,HT	fever	98(4l)	6	19.4	10.4	289	25	18	0.83	74
S	74	M	8	none	cough/fever	94(2l)	9	8.2	19.3	509	92	87	0.88	65
S	66	M	5	HT,COPD,cancer	cough/fever	92→88(2l)	4.8	18.8	2.54	325	34	34	0.87	68
S	67	M	4	DM,COPD,obs	fever	89	3.5	19	10.2	377	131	97	0.73	82
S	67	M	13	none	fever	98(4l)	6.7	26.4	5.8	406	87	46	1.7	32
S	69	M	4	none	cough	92(4l)	12.3	5.2	11.5	597	87	163	1.57	35
S	81	M	5	none	cough	91(6l)	9.6	7.1	17.5	431	40	20	0.8	70
S	62	M	8	HT,DM	cough/fever	92(8l)	6.3	12.2	9.67	552	43	22	0.73	84
S	84	M	10	DM,HT	fever	96(7l)	8	5.9	8.47	699	54	49	0.71	79
S	81	M	5	none	fever	92	5.3	8.5	26	311	37	24	0.69	82
S	71	M	6	HT,CRF,cancer	cough/fever	83(15l)	8.7	22.5	20.1	513	43	32	2.34	22
S	62	M	14	HT,COPD,obs	cough/fever	95(intubation)	7.8	5.5	24.2	1136	92	59	0.84	72
S	42	F	10	obs	fever/diarrhea	93(10l)	7.8	15.6	22.3	834	60	85	0.52	100
