# Supplementary material for: Quantitative analysis of chest computed tomography of COVID-19 pneumonia using a software widely used in Japan
Source: PLoS One. 2023 Oct 23;18(10):e0287953. doi: 10.1371/journal.pone.0287953 (PMC10593239; doi:10.1371/journal.pone.0287953)
Supplement: S1 File — (PDF) [file pone.0287953.s002.pdf]

PVR (%) by Z2

No./HU	-500	-550	-600	-650	-700
1	1.79	4.06	6.72	10.35	15.36
2	4.32	5.87	9.14	13.37	19.59
3	7.35	9.02	13.47	18.58	26.36
4	3.15	5.55	8.98	13.03	17.78
5	0.47	3.53	6.64	12.32	22.8
6	23.23	26.67	38.84	51.09	66.51
7	57.44	49.61	64.45	69.81	77.13
8	4.69	6.2	9.65	13.62	18.71
9	12.85	15.47	18.55	22.56	27.98
10	10.5	12.66	18.25	22.06	26.39

CT score by Z2

No./HU	-500	-600
1	3	5
2	5	5
3	5	6
4	3	5
5	5	5
6	7	10
7	15	15
8	5	5
9	5	7
10	5	6
