# Supplementary material for: Quantitative analysis of chest computed tomography of COVID-19 pneumonia using a software widely used in Japan
Source: PLoS One. 2023 Oct 23;18(10):e0287953. doi: 10.1371/journal.pone.0287953 (PMC10593239; doi:10.1371/journal.pone.0287953)
Supplement: S2 File — (PDF) [file pone.0287953.s003.pdf]

PVR and CT score
by Z2 of 53 cases

sevirety/PVR, CT score	whole lung (PVR)	left lung	left upper	left lower	right lung	right upper	right middle	right lower	CT score
M	3.76	5.72	2.56	10.2	2.07	1.8	1.8	2.4	6
M	3.5	5	3.57	8.68	2.3	1.62	1.57	3.17	6
M	3.32	2.49	2.08	2.8	4.06	1.9	1.31	6.29	5
M	6.81	9.49	7.7	11.1	5.06	4.82	3.54	5.9	6
M	8.26	6.64	3.23	11.73	9.54	2.83	3.46	21.4	7
M	2.25	2.25	1.91	2.76	2.26	2.17	1.76	2.56	5
M	19.88	24.53	10.04	60.04	16.06	7.19	4.02	34.24	12
M	13.8	16.02	10.54	43.64	11.95	7.47	5.87	34.98	12
M	14.21	17.31	6.11	45.64	11.88	4.86	2.26	30.76	10
M	6.63	8.18	7.03	100	5.57	3.41	99.99	7.05	15
M	3.61	3.92	4.26	3.44	3.34	4.41	1.83	3.74	5
M	5.9	7.28	3.52	13.44	4.77	2.23	2.36	9.23	7
M	2.33	2.17	1.75	2.56	2.5	2.28	1.87	3.02	5
M	6.96	3.24	2.34	4.05	10.31	6.84	1.77	18.22	7
M	2.33	2.77	1.81	3.58	1.97	1.64	1.81	2.21	5
M	1.63	1.71	1.61	1.79	1.55	1.55	1.49	1.58	5
M	1.73	1.89	1.54	2.27	1.59	1.45	1.63	1.69	5
M	4.32	5.67	2.54	9.86	3.13	2.32	1.89	4.53	6
M	7.42	6.68	3.38	11.88	8.06	4.34	4.61	13.93	7
M	8.68	5.257	3.62	7.97	11.61	5.79	3.09	22.26	8
M	10.53	11.62	3.82	31.57	9.66	2.28	2.66	23.07	8

M	1.72	1.77	1.63	1.91	1.68	1.71	1.5	1.74	5
M	40.11	36.88	31.99	42	42.84	48.38	13.6	51.17	15
M	14.75	12.08	10.3	14.58	16.75	12.35	7.26	25.1	11
M	18.53	10.09	6.01	14.41	29.02	20.85	17.07	45.14	11
M	1.87	2.04	2.12	1.97	1.73	1.7	100	1.71	9
M	15.15	14.26	4.19	40.36	15.88	8.85	2.61	53.49	11
M	8.47	10.95	6.57	19.09	6.55	3.12	1.82	12.25	8
M	3.06	3.58	2.36	5.53	2.63	2.46	1.72	3.49	6
M	1.84	1.58	1.83	1.39	2.12	2.65	2.33	1.72	5
M	3.6	3.06	2.36	4.53	4.02	1.85	1.72	6.83	6
M	10.5	12.2	7.07	18.24	9.17	6.81	1.85	17.3	9
M	8.65	8.76	5.66	12.366	8.56	2.5	2.42	18.94	8
M	2.35	2.58	2.18	3.07	2.18	1.97	2.01	2.41	5
M	7.63	7.23	5.45	11.32	7.91	3.92	99.77	4.7	11
M	2.87	2.59	2.03	3.69	3.08	2.47	1.54	5.71	6
M	6.82	12.71	5.22	99.84	4.13	2.97	2.83	6.85	11
M	4.81	6.22	3.38	10.38	3.78	3.53	2.5	4.45	6
S	8.41	15.12	8.84	25.76	3.37	3.13	1.61	4.2	8
S	14.88	14.672	9.632	31.736	13.376	10.824	7.224	26.8	12
S	8.85	4.92	4.46	5.47	12.86	7.33	5.56	22.72	9
S	15.06	8.93	4.05	14.91	22.8	11.91	6.4	48.38	10
S	3.32	2.39	2.26	2.55	4.19	7.02	1.86	2.83	6
S	6.48	9.52	8.9	10.51	4.26	3.3	1.7	6.96	8
S	18.88	29.87	20.94	46.55	11.31	4.37	2.44	27.42	10
S	9.66	16.25	9.38	25.39	3.76	1.34	2.28	6.35	9
S	41.52	39.27	32.44	48.85	43.35	50.12	12.52	51.57	16

S	43.01	60.91	50.1	75.26	31.22	28.99	5.54	53.05	18
S	40.16	37.14	24.88	68.19	42.48	28.29	41.54	62.89	16
S	22.67	30.65	30.94	30.45	17.73	30.86	10.1	10.38	13
S	35.99	29.34	18.35	56.93	41.34	40.94	13.34	66.17	15
S	38.54	53.37	52.99	98.52	27.91	8.25	99.43	15.14	18
S	63.26	66.54	69.91	60.79	60.6	57.41	56.81	63.48	20
