# Supplementary material for: Quantitative analysis of chest computed tomography of COVID-19 pneumonia using a software widely used in Japan
Source: PLoS One. 2023 Oct 23;18(10):e0287953. doi: 10.1371/journal.pone.0287953 (PMC10593239; doi:10.1371/journal.pone.0287953)
Supplement: S3 File — (PDF) [file pone.0287953.s004.pdf]

Manual PVR and CT score by two radiologists (A,B)

No./PVR, CT score	Rt. lesion(A)	Rt. Lung(A)	Lt. lesion (A)	Lt. lung (A)	PVR (A)	CT score(A)	Rt. lesion (B)	Rt. Lung (B)	Lt. lesion (B)	Lt. lung (B)	PVR (B)
1	13482	187850	13373	162912	7.66	5	11623	213701	8949	162972	5.46
2	29410	162895	8171	145946	12.17	8	17595	170291	8584	158168	7.97
3	25644	130571	11445	106299	15.66	10	13024	104213	6495	78855	10.66
4	30720	224004	38500	178082	17.21	13	32682	226583	39893	181636	17.78
5	1166	117744	1335	77212	1.28	5	1213	120623	1239	82649	1.21
6	29577	73172	23447	71849	36.56	18	18777	90139	18043	78341	21.85
7	65920	103914	53884	70380	67.74	20	57648	105581	47067	83730	55.31
8	41048	164776	19296	165787	18.25	16	33061	171521	17576	168514	14.89
9	25223	121715	19842	108395	19.58	11	22150	129199	17133	113451	16.19
10	58700	136580	23910	168862	27.05	15	46050	142987	18814	177162	20.26

CT score(B)
8
9
10
12
6
17
20
12
11
15
