# Supplementary material for: Quantitative analysis of chest computed tomography of COVID-19 pneumonia using a software widely used in Japan
Source: PLoS One. 2023 Oct 23;18(10):e0287953. doi: 10.1371/journal.pone.0287953 (PMC10593239; doi:10.1371/journal.pone.0287953)
Supplement: Attachment — Submitted filename: Manuscript Number PONE-D-23-17972.docx [file pone.0287953.s005.docx]

Summary.

The authors have conducted a retrospective analyses of conventional 5mm axial CT scan interpretation by trained radiologists and compared their qualitative studies to the ability of a quantitative software in determining patient pneumonia outcomes n patients with COVID pneumonia.

Strengths:

The study reported has appropriate statistical power and is an important study in terms of the value that quantitative studies can lend to predicting patient clinical outcomes.

The authors correctly point out the objective and added value of having such quantitative metrics: one being reducing the time consumption and error in manually tracing the images ( slice by slice) and doing image segmentation.

Weaknesses:

BUT, although the authors demonstrate the improved ability to quantify the pneumonia, their statement pointing to the ineffectualness of the qualitative report is not fair and is based on comparing apples to oranges with too many confounding variables. There are one main methodological confound embedded in their analyses and a conceptual error of the radiologist’s role in patient care that leads to an erroneous conclusion at the end of their manuscript:

a. The radiologists were reviewing lower resolution CT scans where the software had thin section CT scans as inputs. If the authors are interested in comparing the ability of radiologist to provide accurate readings, they should ask the radiologist to interpret high resolution pulmonary CT (HRCT) and compare that to the performance of the software. In so doing, at least the start points ( in terms of spatial resolution) are comparable.
b. There seems to be a misunderstanding of what it is that the radiologist is supposed to be doing. Radiology is a clinical medical consultation and not a lab test. It is by its very nature interpretative and qualitative. Quantification is a tool that can be used by the radiologist to further enhance his/her ability to provide greater detailed interpretation of the underlying pathophysiology that is occurring in the patient and to use this understanding to propose adding imaging that could further help the referring physician what is happening to the patient.
c. The radiologic test itself is NOT the end product. So, the author’s conclusions that the software can be used ***in lieu*** of the radiologic interpretative report is not quite correct. The data show that using quantitative analyses can increase the ability of the radiologist to add valuable information into his or her report.

Recommendation: major revision to address the points raised above.
