# Supplementary material for: Quantitative analysis of chest computed tomography of COVID-19 pneumonia using a software widely used in Japan
Source: PLoS One. 2023 Oct 23;18(10):e0287953. doi: 10.1371/journal.pone.0287953 (PMC10593239; doi:10.1371/journal.pone.0287953)
Supplement: Attachment — Submitted filename: response to reviewer 2.docx [file pone.0287953.s006.docx]

**Response to Reviewer 2**

Thank you very much for your comments, which have helped us improve the quality of our work.

We thank you for acknowledging that the statistical power of our study was adequate and that quantitative studies are useful in predicting clinical outcomes in patients.

As you pointed out, we did not disclose the research data. We will improve the quality of our work and attach measurement data to the extent that they can be clearly stated.

Moreover, please note that the purpose of this study was not to compare quantitative evaluations made by radiologists and image analysis software (Z2), but to explore the repurposition of the image analysis software (Z2), which is widely used in Japan and was originally intended to evaluate emphysema, for the assessment and potential classification of COVID-19 pneumonia severity.

The Z2 software was released in 2010 and is currently applied in approximately 300 facilities in Japan.

It can automatically extract the bronchi, lungs, and low-attenuation areas, and can measure the volumes of the entire lung and low-attenuation areas. The area below the threshold density of the lung field is defined as the low attenuation area of the emphysema, and the low attenuation volume is measured for the evaluation of COPD.

In this study, we used Z2 to measure the lung field with a threshold concentration or higher as pneumonia. As Z2 has not been used to evaluate lesions above a certain concentration, such as pneumonia, it was necessary to determine an appropriate threshold in Hounsfield units (HUs). (lines 79–87)

Therefore, for each of the 10 selected cases, an appropriate threshold was determined by comparing the PVR measured manually by two radiologists (lines 118–121), and the PVR was measured with Z2 under different conditions in the range of -500 HU to -700 HU. (lines 135–137)

Undoubtedly, we do not believe that quantitative evaluation alone is sufficient. Two radiologists also performed a qualitative evaluation of CT findings (GGO, crazy-paving, infiltrative shadows, emphysema) in 53 cases (lines 118–119), and the results were consistent with those of previous reports. In particular, there was a significant difference in the clinical severity of the presence of consolidations (lines 331–335). Although not mentioned, both radiologists used both 5-mm slice images and high-resolution CT (HRCT) images for the macroscopic qualitative evaluation of 53 cases.

We believe that determining the appropriate conditions of Z2, originally intended for evaluating emphysema, for evaluating COVID-19 pneumonia is a task that requires the perspective of a radiologist.

In addition, Z2 may not recognize the boundaries between lung field infiltration shadows, atelectasis, and extrapulmonary structures, such as pleural effusions and chest walls; therefore, it is not enough to simply measure automatically by a computer, and therefore, corrections made by a radiologist are needed. (lines 139–144)

We apologize but we did not clearly understand the reviewer 2’s comment on “comparing apples and oranges.” Is this a reference to the comparison between the PVR by Z2 and CT scores by radiologists? If so, there is a misunderstanding and we apologize for the confusion. We compared the PVR with Z2 and CT scores with Z2. The purpose of comparing the two is as follows.

The CT score visually scores each lobe of the lung in 25% increments, and although it is simple and versatile, it is subjective and cumbersome. In contrast, the PVR is automatically measured using software and AI. Thus, objective and accurate results are available quickly.

To measure PVR macroscopically, manual tracing using a slice-by-slice method can be considered, as was done for the 10 cases selected in this study, but this is an effortful and time-consuming process, which is not ideal for clinical practice.

If automatic measurement is possible, it is obvious that it is more accurate to use the PVR rather than the CT score. In this study, the AUC of the ROC for severity classification using PVR and the CT score of Z2 showed that the PVR results exceeded the CT score (lines 247–251).

However, the problem with automatic measurements of PVR is that various software and AI tools measure it using different algorithms. Thus, the results cannot be treated as equivalent. (lines 309–314) Therefore, in fact, past studies on automatic quantitative analysis of chest CT scans of COVID-19 pneumonia have evaluated the PVR by converting it into the CT score (References #11, 17, 18).

In this regard, Z2 is a software that is widely used in Japan; thus, new analysis approaches using Z2 could be easily implemented and would be readily available. (lines 310–314)

We would like to revise the corresponding part in the manuscript to make it easier to understand the benefits of using software that is already on the market rather than using new software or AI.
